# Designing Allele-Specific Competitive-Extension PCR-Based Assays for High-Throughput Genotyping and Gene Characterization

**DOI:** 10.3389/fmolb.2022.773956

**Published:** 2022-03-01

**Authors:** Ruslan Kalendar, Alexandr V. Shustov, Ilyas Akhmetollayev, Ulykbek Kairov

**Affiliations:** ^1^ Institute of Biotechnology HiLIFE, University of Helsinki, Helsinki, Finland; ^2^ PrimerDigital Ltd., Helsinki, Finland; ^3^ National Laboratory Astana, Nazarbayev University, Nur-Sultan, Kazakhstan; ^4^ National Center for Biotechnology, Nur-Sultan, Kazakhstan

**Keywords:** genotyping assay design software, polymerase chain reaction-based markers, diagnostic system, genotyping system, single nucleotide polymorphism, insertion-deletion polymorphism

## Abstract

Polymerase chain reaction (PCR) is a simple and rapid method that can detect nucleotide polymorphisms and sequence variation in basic research applications, agriculture, and medicine. Variants of PCR, collectively known as allele-specific PCR (AS-PCR), use a competitive reaction in the presence of allele-specific primers to preferentially amplify only certain alleles. This method, originally named by its developers as Kompetitive Allele Specific PCR (KASP), is an AS-PCR variant adapted for fluorescence-based detection of amplification results. We developed a bioinformatic tool for designing probe sequences for PCR-based genotyping assays. Probe sequences are designed in both directions, and both single nucleotide polymorphisms (SNPs) and insertion-deletions (InDels) may be targeted. In addition, the tool allows discrimination of up to four-allelic variants at a single SNP site. To increase both the reaction specificity and the discriminative power of SNP genotyping, each allele-specific primer is designed such that the penultimate base before the primer’s 3′ end base is positioned at the SNP site. The tool allows design of custom FRET cassette reporter systems for fluorescence-based assays. FastPCR is a user-friendly and powerful Java-based software that is freely available (http://primerdigital.com/tools/). Using the FastPCR environment and the tool for designing AS-PCR provides unparalleled flexibility for developing genotyping assays and specific and sensitive diagnostic PCR-based tests, which translates into a greater likelihood of research success.

## Introduction

High-throughput technologies for nucleotide sequence analysis and detection of sequence variation have been increasingly used for plant and animal genotyping, forensics, genetic medicine, and other fields of genetic testing. The most frequently encountered sequence variations in any genome are single-nucleotide polymorphisms (SNP) (Clevenger et al., 2015). The primary consideration for selecting a marker type for genotyping is the information content of the polymorphisms and the informative capacity of the test. In this regard, SNPs are very effective markers. A variety of SNP genotyping methods are available, and new methods appear regularly with the aim of reducing cost and increasing throughput. PCR can be adapted for rapid detection of single-base changes in genomic DNA by using a family of closely related methods, such as allele-specific PCR (AS-PCR) (Ugozzoli and Wallace, 1991; Bottema and Sommer, 1993; Spierings et al., 2006), PCR-amplification of specific alleles (PASA) (Sommer et al., 1989; Sarkar et al., 1990), allele-specific amplification (ASA), and amplification refractory mutation system (ARMS) (Newton et al., 1989; Nichols et al., 1989; Wu et al., 1989). All these methods use specifically designed PCR-primer sets containing allele-specific primers (ASP) in which the allele specificity is determined by the base at or near the 3′ end. In principle, AS-PCR assays can be developed to analyse almost any allelic variation (Sommer et al., 1989; Ye et al., 1992).

There currently exists an enormous number and variety of established methods applicable for SNP analysis and genotyping based on distinctly different platforms and approaches (Kim and Misra, 2007). FRET (Fluorescence Resonance Energy Transfer) is one such method and is based on dual fluorescence that is quantified during ligation. Another method is allele-specific PCR with SNP targeting (Didenko, 2001; Kaur et al., 2020). Single-plex PCRs for SNP analysis, such as TaqMan (Life Technologies, USA) and SimpleProbe (Roche Applied Science, USA) have been described as valuable additions for marker-assisted selection in plant breeding (Makhoul et al., 2020; Della Coletta et al., 2021). Further improvements for AS-PCR include using fluorescence to detect the amplification of a specific allele. Kompetitive Allele Specific PCR (KASP) (LGC Biosearch Technologies, Teddington, UK) and PCR Allele Competitive Extension (PACE) (Integrated DNA Technologies, Inc.) genotyping represent developments to the original AS-PCR approach. These techniques use fluorescence resonance energy transfer (FRET) for signal generation and both allow accurate bi-allelic discrimination of known SNPs and insertion-deletion polymorphisms (InDels). The KASP method has great potential for expanded utilization, as it requires only a slight modification to commonly used procedures for designing ASPs, offers convenient FRET detection, and master-mixes are commercially available (Wang et al., 2015; Ryu et al., 2018; Brusa et al., 2021). In KASP, PCR amplification is performed using a pair of allele-specific (forward) primers and a single common (reverse) primer. In addition to the common-core primers, the reaction mixture is supplemented with a FRET cassette. This is a duplex of two synthetic complementary oligonucleotides; one is labelled with a fluorescent dye and the other carries a fluorescence quencher. Further, each ASP has a unique 5′ terminal extension (tail), which is complementary to the sequence in the FRET cassette. The oligonucleotides in the FRET cassette are modified such that they do not participate in polymerase-mediated extension steps. The dye-labelled oligonucleotide is capable of annealing to the reverse-complement of the tail sequence in PCR fragments containing one selected allele. During amplification of the allele with participation of the tailed ASP, an amount of DNA increases to which the dye-labelled component anneals. This disrupts the integrity of the FRET cassette and the fluorescent dye is spatially separated from the quencher and thus able to emit fluorescence. Unlike other PCR-based genotyping assays, KASP/PACE requires no labelling of the target-specific primers/probes, which provides additional flexibility in the assay design. Both methods use two reporting cassettes. If a genotype at a given SNP is homozygous, only one of the two possible fluorescent signals will be generated. If the genotype is heterozygous, both fluorescent signals will be generated. KASP/PACE technology is especially suitable for high-volume screening projects, such as in plant breeding. KASP/PACE technology has a key feature, which is utilizing a universal FRET cassette reporter system that eliminates the need for dual-labelled probes. Commercial companies produce PCR additives, called master mix, which contains one or more ‘universal’ FRET cassette(s). In theory, this additive can be used to upgrade existing AS-PCR assays to KASP/PACE, provided that new ASPs are used that have the described cassette-specific tails. For example, a protocol upon which this work is based, uses chemistry consisting of the following two parts. These are the assay mix (template-specific, contains target DNA, two ASPs [for binary SNP] and a single reverse primer) and master mix (not template-specific, combines all reagents required for PCR and in addition contains two different FRET cassettes, one cassette labelled with FAM dye and the other with HEX dye). Each ASP carries a unique tail sequence that corresponds to a FRET cassette. In other FRET AS-PCR methods, such as Amplifluor (Rickert et al., 2004; Fuhrman et al., 2008) and STARP (Rasheed et al., 2016; Long et al., 2017; Li et al., 2019; Wu et al., 2020), these two parts are completely separated into non-labelled ASPs such that the last base of the primer’s 3′ end base is positioned on the SNP site, and labelled universal probes (UPs) that carries a fluorophore at the 5′ terminus and a quencher attached in the middle of the universal probes (Nazarenko et al., 1997). Simple and inexpensive ASPs can be designed and ordered for each SNP separately, while the relatively expensive UPs with fluorophores and quenchers are ordered just once for a stock that can be used over a very long time in many different SNP analyses. The principle of ASP-UP is similar to that of Molecular Beacons, with the addition of specialized identical “tags” at the 5′ end of the ASP and the 3′ end of the UP. In this particular case, ASPs are slightly longer (Myakishev et al., 2001). In KASP-related methods, a set of non-labelled ASPs includes two forward primers and a single common reverse primer that act on a competitive basis in conjunction with one of two corresponding UPs with “hair-pin” FRET structures that end with either FAM or HEX/VIC fluorophores. This approach allows for great flexibility in assay design, which translates into a higher overall success rate for SNP genotyping and detection of InDels. This principle of separated ASPs and UPs is used in various methods, including commercially produced Amplifluor (Merck KGaA) and KASP markers (LGC Biosearch Technologies) for fluorescent signal generation that enable bi-allelic discrimination and genotyping of SNPs or InDels.

Developing high-throughput, multiplex genotyping assays require using computerized approaches to design primers and probes and select reaction conditions. In recent years, several software tools have been developed to aid AS-PCR assay development, including GSP (Wang et al., 2016), PrimerSearch-EMBOSS (http://emboss.open-bio.org/rel/rel6/apps/primersearch.html), WASP (Wangkumhang et al., 2007), PolyMarker (Ramirez-Gonzalez et al., 2015), KASPspoon (Alsamman et al., 2019), and PUNS (Boutros and Okey, 2004). However, not all of these programs are readily available or broadly applicable; some are no longer actively updated whilst others are extremely narrow in their applicability.

Here we describe a software tool and a modified KASP method that further increases the convenience and power of the genotyping protocol. We have named this allele-specific quantitative PCR (ASQ). The software facilitates the development of assays using KASP and PACE technology and works with SNP and InDel polymorphisms. For all polymorphisms taken into the study, the program produces the thermodynamically optimal combination of ASPs for single-plex or multiplex assays. During ASP design, a user can add a tail sequence (which can be a custom sequence) or a sequence that matches a commercially available aster mix (e.g., manufactured by LGC Biosearch Technologies, or Merck KGaA). If a commercial FRET cassette is not optimal, the program allows use of custom FRET cassettes. The program also computes the optimal reaction conditions to perform KASP. This software will be most useful for multiplex genotyping in a high-performance environment. Furthermore, potential uses are not limited to genotyping. The program can produce tests for selecting mutants, e.g., after genome editing or gene knockout (Lee et al., 2016). Other possible applications include analysis of genetic variations in microorganisms, strain identification, detecting genetic markers of resistance or virulence, and tracing pathogens in epidemiology and medical diagnostics. The ASQ method described here is a tool for assessing the relative amount of different allelic variants in a sample. This method can be useful for a variety of tasks. For example, ASQ can be used to measure a fraction of admixed GMO material in samples of agricultural products or to measure a portion of malignant cells in samples from cancer patients. Distinctive features of the presented program make it unique in its class. These features include computing of multiplex reactions for simultaneous identification of up to four alleles (e.g., 3-state or 4-state polymorphisms); use of input polymorphisms of mixed type (overlapping SNP and InDel sites); and absence of restrictions on the length difference between InDel alleles during genotyping of InDels. The program automatically calculates primers for possible amplification in both directions from the polymorphic site. The program allows the user to include in the protocol commercially available master mixes and FRET cassettes or to create unique FRET cassettes and use custom reagents in designing other assays. The software described herein is integrated into and works inside the FastPCR environment. FastPCR is an integrated software space for developing PCR-based processes. The FastPCR program, including the presented AS-PCR-designing tool, may be used online or downloaded and used in Microsoft Windows (Kalendar et al., 2011; Kalendar et al., 2017) and is freely available at http://primerdigital.com/tools/. This AS-PCR tool simplifies and automates design of genotyping assays, resulting in a greater likelihood of success.

## Materials and Methods

### Selection of Highly Informative SNP Candidates

SNP data in the SNPforID browser (http://spsmart.cesga.es/snpforid.php) were employed in the present study for SNP genotyping of humans in forensic studies. The allelic frequencies were used for screening to select highly informative SNP candidates. As markers with even allelic distributions have high observed heterozygosity and are more informative, 7 of the SNPs were selected with a common 40:60-60:40 allelic distribution in European and Central Asian populations. All of the selected markers are located on autosomal chromosomes (Supplementary Table S1). The marker also known as “C/T(-13910)” located in the MCM6 gene but with influence on the lactase LCT gene (rs4988235) is one of two SNPs that is associated with the primary haplotype associated with hypolactasia, or more commonly known as lactose intolerance in European populations.

### Sample Preparation

This work was discussed by the institutional review board and was approved by the ethical committee of the Center for Life Sciences, National Laboratory Astana, Nazarbayev University (protocol #21, 10 October 2017). Institutional written informed consent about nationality declaring, DNA extraction and for further investigation was signed and obtained from the participated individuals.

Informed consent was obtained for each participant. DNA was extracted using QIAamp DNA Blood Mini Kit (Qiagen, Hilden, Germany). The extracted DNA pellet was diluted in TE buffer (10 mM Tris pH 8.0, 0.1 mM EDTA), and DNA concentration was measured with a NanoDrop spectrophotometer (Thermo Fisher Scientific, USA). Quality was assessed using 1 mg of DNA visualised after running in a 1% agarose gel.

### Real-Time PCR Analysis

A QuantStudio-7 Real-Time PCR instrument (Thermo Fisher Scientific, USA) and CFX96 Real-Time PCR Detection System (Bio-Rad, USA) were used. These instruments have detection systems with filters for FAM, VIC/HEX, Cy3, Cy5, and ROX fluorophores. While SNP identity calls were made automatically using software accompanying the instruments, amplification curves were checked for each genotype manually for final allele discrimination. SNP genotyping experiments used at least three to eight biological replicates and were repeated three times.

The PCR conditions employed an altered PCR cocktail composition (Table 1). PCR plates with 96-wells were used with a 15 μl total reaction volume in each well. The PCR mix consisted of the following reagents: 1x OneTaq buffer (total 3 mM MgCl_2_), 0.2 mM of each dNTP, 0.2 μM of each UP, 0.5 μM quencher oligo, 0.1 μM of each AS primer, 0.3 μM of reverse primer, and 0.5 units of Taq DNA polymerase (NEB). Half of the PCR volume was genomic DNA, adjusted to 5 ng/μl.

**TABLE 1:** PCR cocktail mix composition for the proposed ASQ method of SNP genotyping.

Component	Concentration	Volume (µl)	Final concentration
5x OneTaq Buffer (with 9 mM MgCl_2_)	5×	20	1×
MgCl_2_	25 mM	4.8	1.2 mM
DNTP	10 mM	2	0.2 mM
ASP-F1	5 µM	2	0.1 µM
ASP-F2	5 µM	2	0.1 µM
ASP-R	5 µM	6	0.3 µM
UP-FAM	10 µM	2	0.2 µM
UP-HEX	10 µM	2	0.2 µM
Uni-Q	50 µM	1	0.5 µM
Taq DNA Polymerase	5 units/µl	0.8	0.04 units/µl
Milli-Q water		37.4	
DNA template	10 ng/μl	20	2 ng/μl
Total		100	

The PCR program was optimised and consisted of 95°C, 120 s; 10 cycles of 10 s at 95°C; 20 s at 55°C; 20 s at 68°C; 30 cycles of 10 s at 95°C; 30 s at 68°C; 30 s at 55°C (Table 2). Fluorescence was monitored during the last step at a second annealing. ASQ was performed to simultaneously detect two alleles in a single tube. Each well was examined for the characteristic fluorescent emissions of both fluorescein (FAM channel) and HEX (HEX channel).

**TABLE 2:** PCR protocol for genotyping using advanced ASQ method for SNP genotyping.

Step	Temperature	Duration (sec)	Notes
1	95°C	120	Initial denaturation
2	95°C	10	First-round denaturation
3	55°C	20	First-round annealing
4	68°C	20	First-round extension
5			10 cycles repeat for steps 2–4
6	95°C	10	Second-round denaturation
7	68C	30	Second-round annealing and extension
8	55°C	30	FRET cassette annealing and signal read
9			30 cycles repeat for steps 6–8

Genotyping with SNP calling was determined automatically. Each experiment was repeated twice and technical replicates confirmed a confidence of SNP calls.

### KASP Assay Design Method

Standard KASP allows genotyping of only two alternative alleles at any specific site. This is because there are two only FRET cassettes, and thus two fluorochromes, present in commercially available kits. However, advances in real-time PCR equipment allow detection of up to 6 spectrally separated dyes.

**FIGURE 1:**
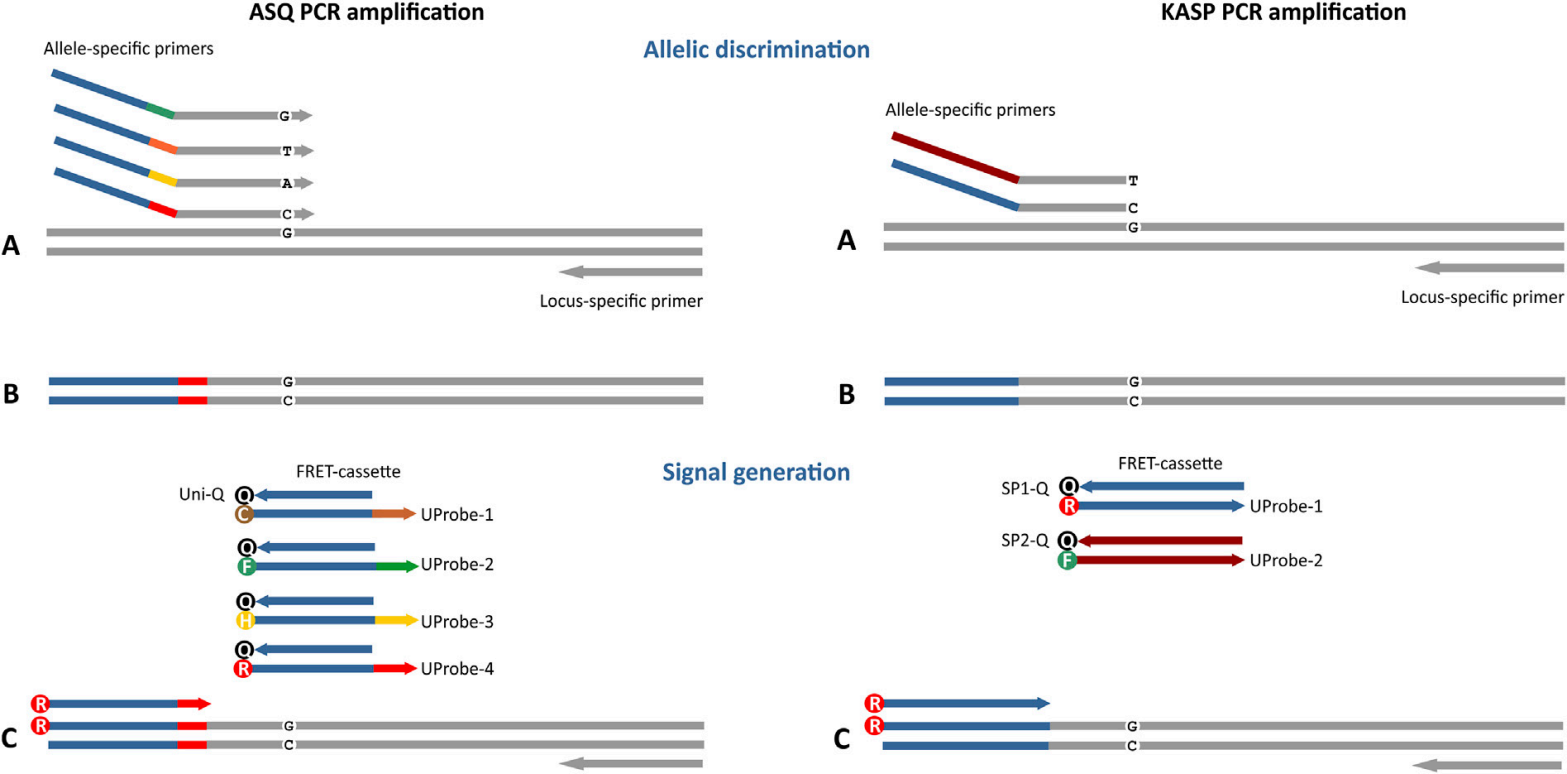
Four-plex fluorescent ASQ assay genotyping system compared to the standard two-plex KASP technology (LGC Biosearch Technologies). The main differences in these approaches are associated with the potential number of simultaneously detectable polymorphic sites (4 in ASQ, 2 in KASP) and the structure of the primers that compose the FRET cassette. For ASQ, the FRET cassette consists of 2 or more of allele-specific primers (ASP) and a fluorescently labelled universal probe (UP) with a single universal quencher probe (Uni-Q). Differences in the tail sequence ASPs and UPs are determined by a unique 6-nt barcode sequence that is not part of the universal tail of the Uni-Q sequence. KASP technology includes 2 variants of ASPs and fluorescently labelled UPs (UP-1/2) with each UP requiring a specific quencher (SP1/2-Q). In addition, the ability to design an allele-specific primer with the SNP site at the penultimate or antepenultimate 3′ base of each allele-specific primer is characteristic of the ASQ method. **(A)** Both allele-specific primers query the SNP locus. Denaturation of DNA template and annealing ASP to the target, PCR round 1. **(B)** Formation of a PCR product containing a specific tail sequence that is complementary to allele-specific primers. This PCR product will be used in subsequent PCR cycles as a template for amplification using a specific fluorescently labelled UP **(C)**. During the first two amplification cycles, a tail sequence is incorporated into the amplicon that is subsequently recognized by a universal, probe-based reporter system.

**FIGURE 2:**
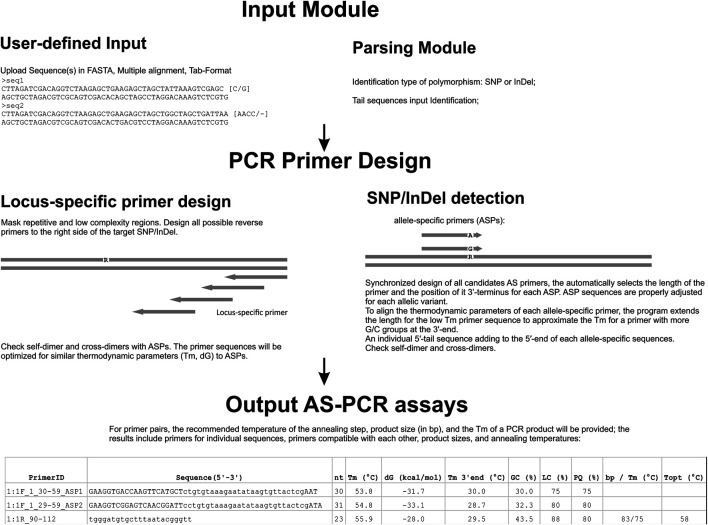
Flowchart of the main steps in the AS-PCR process. Data can be uploaded into FastPCR, which accepts a single sequence or multiple separate DNA sequences in FASTA, tabulated format, multiple alignment, or from clipboard. SNP sequence data are pre-processed by FastPCR prior to execution to collect the SNP position and also to remove non-DNA symbols from the entire sequence. The user sets the various parameters for primer design for the DNA or SNP sequences, such as primer maximum and minimum lengths and *T*_*m*_. Repetitive or low-complexity sequences are excluded from primer design by default (this can be changed by the user). The initial primer design attempts both directions and a reverse primer is designed at the upstream accordingly. An allele-specific primer with the SNP site is designed at the second (penultimate) position of the primer’s 3′ terminus or to any other position in manual or automatic mode. Once the allele-specific primers are designed, an individual 5′-tail sequence is added to the 5′ end of each allele-specific sequence. This combined whole primer sequence is reanalysed for self- and cross-dimerization. Finally, results are returned to the text-editor window.

In addition, there are approaches to overcome physical spectral dye channels and expand the potential of spectral channels to practically detect an unlimited number of independent targets (Marras et al., 2019). Theoretically, the number of different multiplex targets or alternative alleles that can be identified in a screening assay can be increased significantly by utilizing a unique combination of two colours for the identification of each target allele (Marras et al., 2019). The novel tool described here allow detection of up to 4 spectrally separated dyes (Figure 1). Moreover, the program calculates even more assay possibilities because the ASPs are searched for on both strands and at both sides from a polymorphism site (Figure 2). The user has the possibility to use the single most suitable set of primers from the output or, if desired, alternative sets of primers may be used to target the same site. The tool also allows design combinations of tail sequences to create a multiplex PCR targeting several different polymorphic sites simultaneously. There is a need to incorporate flexibility in a primer-designing algorithm to select AS primers, as placing the 3′-terminal nucleotide on the polymorphic site may not be an optimal solution for some sites. Briefly, to achieve optimal amplification, the tool automatically selects the primer length and the position of its 3′ terminus for each ASP. This kind of optimization is also necessary when working with InDels. For example, for a null-site polymorphism (i.e., one or more nucleotide[s] absent in one allele or there are insertions in other alleles), the ASP sequence to target this allele will be positioned such that the primer’s 3′-terminal sequences are sufficient for annealing. Thus, ASP sequences are properly adjusted for each allelic variant. In case of short InDels (<4 nucleotides), the better solution is to individually design the sequence of the ASP 3′ termini for each variant, which can be achieved with this tool. Thus, the algorithm allows the generation of ASPs with 3′-terminal sequences that are unique for each allelic variant. This tool works within the FastPCR environment, which contains numerous functions that are necessary for the development of PCR primers for most applications. These include complex PCR designs, such as multiplex PCR or Loop-mediated Isothermal Amplification (LAMP). Regardless of the intended application, PCR primer design using FastPCR’s algorithms attempts to generate sets of primers with a high likelihood of success. For this purpose, there is an automatic check for unwanted binding sites (non-specific priming control) in input sequences to prevent the generation of side PCR products. Inputs may be linear or circular sequences. Moreover, the FastPCR environment is expandable, allowing addition of specialized functions for specific tasks.

Components of FastPCR are programmed in Java and thus the program is intrinsically multiplatform. Entering inputs and execution and post-processing of the output can be performed with ease. The selection of the optimal target for KASP primer sets is performed in the same way as for PCR primers. The thermodynamic stability of hairpins is evaluated primarily for the 3′-terminal sequence and, if desired, for the primer’s 5′ terminus as well. The user may select thermodynamic options that will be used to design primer sets (within different templates) or individually set the options for each template in a set of several ones. The program calculates several primer pairs or sets from which the user may choose the best. The user can request a desired product size or instruct the software to search for primer pairs for diagnostic PCR, in which case a whole input sequence will be used to search for the best primer pair. By default, the program selects primers sets with compatible melting temperatures. Alternatively, the user may choose primer sets with similar Gibbs free energy (dG). The tool checks for self-dimer and cross-dimer interactions for all primers. Additionally, users may design compatible primer sets for a predetermined primer (probe) or a list of predetermined primers (probes). This feature can be useful when designing a multiplex assay for different targets. FastPCR allows avoiding non-specific amplification by choosing the best paired primer for a given oligonucleotide to achieve the highest likelihood of success.

To this end, the program chooses primers that avoid repeats or other stable motifs, like potential G-quadruplex sequences. Results with program-suggested primers and primer sets may be exported in common exchange formats (e.g., MS Excel). The following parameters may be placed into spreadsheets: primer name, sequence, location on target, sequence direction, length, melting temperature, GC content, molecular weight, molar extinction coefficient, linguistic complexity (LC), and primer quality (PQ).

For primer pairs, the recommended temperature of the annealing step, product size (in bp), and the *T*_*m*_ of a PCR product will be provided. For pairs, the results include primers for individual sequences, primers compatible with each other, product sizes, and annealing temperatures. For all selected primer pairs, the program provides (in tabular form) the compatibility of the two primers in one reaction, including primer-dimers, cross-hybridization, and product size overlaps (Figure 2).

Users will find examples for entering sequences encoding polymorphic sites in the File menu of the FastPCR software. Input sequences can be in FASTA, in tabular formats, or as multiple sequence alignment as given in the examples. Square brackets are used to specify borders of a polymorphic site of interest. The alternative alleles of a SNP are indicated by brackets, e.g., [C/G] (alternative way to code—[S]). Individual allelic variants in a polymorphic site are thus listed as [variant1/variant2] for binary polymorphism or [variant1/variant2/variant3/variant4] for quaternary polymorphism. Alternatively, IUPAC notation can be used for a degenerate base (e.g., FASTA format) (Supplementary material). When a polymorphic site contains InDels, the same format with square brackets ([variant1/variant2/variant3/variant4]; two or more different variants per site) is used to describe individual variants. Only differences between the variants must be shown within the brackets (Supplementary material). The program allows primers to be designed with the SNP placed at any position with respect to the 3’ end. To do this, the user must specify the desired SNP position using the command with the position number. By default, the program automatically specifies the SNP position at a penultimate base (-aspcr). In addition, for InDel sites, the number of discreditable nucleotides at the 3′ end of each ASP can be automatically determined depending on the polymorphism variant. For InDels, the potential of 3′-end generation for each ASP design option is wider, thus offering the possibility of a more effective discrimination of the various alleles. The program will also determine the minimum length of the 3′ end of each ASP for effective detection of all polymorphisms. The user can define the position of the polymorphic site in any of the positions, such as at a first or penultimate or another base in 3′ end of each ASP to increase reaction specificity and allele discrimination. The 3′-end sequence of each ASP should be designed such that each ASP is unique and specific only to its target. The program uses the sequence from the insertion to generate the 3′-end of ASP sequence for a null-site polymorphism. In the case of a single- or two-base InDel, the following sequence after target site will be used to generate the 3′ end of each ASP. The maximum length that the program can use to develop unique 3′ ends of ASP sequences in the program is 12 nucleotides. This should be sufficient for any InDel polymorphism to generate unique sequences for all 3′ ends of ASPs. The primer sequences will be optimized for similar thermodynamic parameters (*T*_*m*_, dG) (Peyret et al., 1999; Bommarito et al., 2000). To align the thermodynamic parameters of each ASP, the program extends the length for the low-*T*_*m*_ primer sequence to approximate the *T*_*m*_ for a primer with more G/C groups at the 3′ end.

## Result

### Design of Universal Probes and Allele-specific Primers and Locus-specific Common Primers

In our experiments, we used four UPs with types of specific tails. Sequences of the four UPs are presented in Table 3; an example of one such ASP for the human SNPs is shown. All SNP-specific primers and UPs labelled with FAM, HEX, Cy3, and Cy5 were synthesized locally (National Center for Biotechnology, Nur-Sultan, Kazakhstan). A stock of each probe and a single quencher (Uni-Q) was prepared by dissolving each in TE buffer. A working stock of all four probes and a quencher was used for assays. A Uni-Q was conjugated with BHQ1 at the 3′ end. The sequence of Uni-Q was complementary to all corresponding probes. The quenchers were shorter in nucleotide length (13 nt) than the UPs (19 nt). The *T*_*m*_ of this quencher oligonucleotide and the complementary probe was 55°C (for oligonucleotide concentration of 500 nM in 55 mM KCl with 2.2 mM Mg^2+^ (3 mM Mg^2+^ minus 0.8 mM dNTP) (Owczarzy et al., 2008) as calculated by FastPCR (Kalendar et al., 2017). The UPs were designed to have a higher *T*_*m*_ (66–68°C). At an annealing-extension temperature of 60–68°C, the AS primers and UPs can bind the target and induce polymerization without much interference from the lower *T*_*m*_ of the quencher oligo. When the temperature is subsequently decreased to 55°C, the Uni-Q binds the tail of the free, single-stranded UPs, but not the double-stranded PCR product. Because the Uni-Q concentration is two- to four-fold higher than that of each UP, most of the free UPs are expected to bind the quencher oligo at 55°C, thus strongly quenching the UP fluorescence. Since the 5′ end of the UP tail is opposite to the 3′ end of the quencher oligo, the interaction is mediated via direct contact-quenching between the 5′ fluorophore and the 3′ quencher present on the tail and quencher oligo, respectively, which for most fluorophores provides stronger quenching than fluorescence resonance energy transfer (FRET) (Fiandaca et al., 2001; Li et al., 2006). The 3′ BHQ1 was used as it has a wide range of absorbance wavelengths and is appropriate for quenching multiple fluorophores simultaneously, including the FAM and Cy5 used for multiplex PCR.

**TABLE 3:** Fluorescent probes (UP) and a quencher oligonucleotide (Uni-Q) for Allele-Specific Quantitative PCR (ASQ).

Primer ID	Sequence (5′-3′)^a^	nt	*T*_*m*_ (°C)^b^	dG (kcal/mole)	GC (%)	LC (%)^c^	Type and fluorescent label
Uni-Q	Accgttcagctgg	13	53.3	−16.7	61.5	100	Universal 3′ Quencher (Eclipse Quencher or 3′ Black Hole Quencher 1)
**Fluorescent probes**							
UP1	cca​gct​gaa​cgg​tAC​GGC​A	19	67.4	−26.4	63.2	86	UP1: 5′-FAM
UP2	cca​gct​gaa​cgg​tCG​TTG​C	19	66.5	−26.4	63.2	95	UP2: 5′-HEX/JOE/VIC
UP3	cca​gct​gaa​cgg​tAG​CCG​A	19	66.9	−26.1	63.2	89	UP3: 5′-Cy3/TAMRA
UP4	cca​gct​gaa​cgg​tGCG​TCA	19	67.9	−26.8	63.2	92	UP4: 5′-Cy5/Liz

a Capital letters used for a unique barcode sequence; underlined letters used for the universal tail in UPs, and Uni-Q.

b Melting temperature (*T*_*m*_) calculated for oligonucleotide concentration of 200 nM in 55 mM KCl with 2.2 mM Mg^2+^.

c Linguistic Complexity (%).

The SNP site in the AS primers of each locus were designed to directly flank the site in both the sense and antisense orientation. The position of the polymorphic site was located in the penultimate base in the 3′ end of each ASP. Amplification primers were designed such that the amplicons ranged in size from 100 to 200 bases and encompassed the SNP site.

The remaining allele-specific region of ASPs was selected to obtain a *T*_*m*_ close to 63°C (range 62–64°C, calculated for oligonucleotide concentration of 200 nM, 55 mM K^+^, in the present of 2.2 mM Mg^2+^ using FastPCR software) (Kalendar et al., 2014). The initial primer design was first attempted for the current strain and then a complement strain direction was attempted and a reverse primer was designed at the upstream accordingly. Once the allele-specific primers were designed, an individual 5′-tail sequence was added to the 5′ end of each allele-specific sequence. This combined whole primer sequence was reanalysed for self- and cross-dimerization.

### AS-PCR Analysis

**FIGURE 3:**
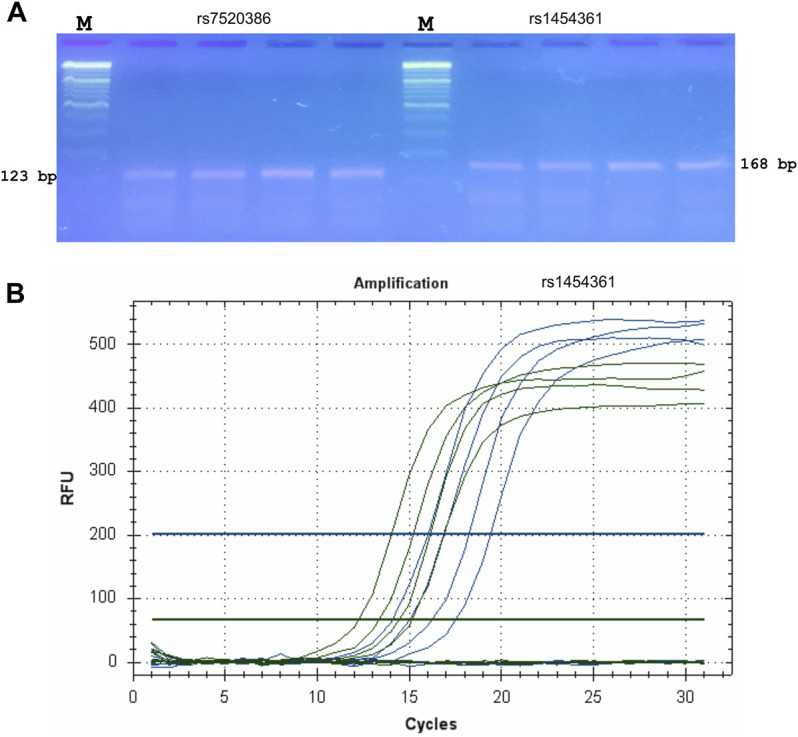
Validation and testing of ASQ method. Probes for ASQ assays were designed using FastPCR to human SNPs (rs7520386 and rs1454361) that were employed for SNP genotyping of humans in forensic studies and PCR products from a series of PCR reactions using these primers were examined by the agarose gel electrophoresis was used without staining **(A)**. M—Thermo Scientific GeneRuler DNA Ladder Mix (100–10,000 bp) stained with SYBR Green I. Primer sequences are displayed in Supplementary Table S1. PCR bands of the correct size (123 bp for rs7520386 and 168 bp for rs1454361, respectively) were obtained from each qPCR. **(B)** qPCR amplification plot for SNP (rs1454361). FAM plot (green) shows amplifications of a A-allele, whereas HEX plot (blue) shows amplifications of a T-allele.

The validation of the proposed ASQ method was performed using SNP for genotyping humans. We designed eight ASQ sets for informative SNP candidates that could be used for genotyping humans and tested their genome specificity using PCR assays. Validation tests were performed for ASQ with quantitative PCR-based experimentation. The corresponding primer sequences are listed in Supplementary Table S1. PCR reactions using primers designed by our tool were performed and the resulting products are presented in Figure 3. In each case, bands of the correct size were obtained from each PCR. These ASQ sets showed genome specificity and identified SNP alleles in the human genome (Supplementary Table S1). SNP allele discrimination for 16 human genotypes is presented in Figure 3. These results for SNP for genotyping of humans validated our conclusion that the proposed ASQ method is very accurate and effective for SNP genotyping; we expect similar results in other animal species. In addition, we developed ASQ sets and applied SNP genotyping in the barley genes HvSAP16 and HvSAP8 (controlling stress-associated proteins); these are validated examples and have been accepted for publication (Baidyussen et al., 2021; Kalendar et al., 2022). Our tool and the ASQ method are suitable for two-, three-, or four-allelic uniplex reactions but can potentially be used for different SNPs in a multiplex format in a range of applications, including medical or forensic studies or other studies involving SNP genotyping.

## Discussion

### Comparison of KASP Assay Design Tool to Existing Software

We compared the functionality of the tool described here with other web-based software available online. Only software programs capable of AS-PCR designs were compared (Table 4). One example of an AS-PCR-directed program is WASP (https://bioinfo.biotec.or.th/WASP) (Wangkumhang et al., 2007). We used example allele sequences published on the WASP website to produce ASPs (primers were computed with WASP and with the KASP/PACE tool) (Supplementary Data S2). It appeared that the primer design in WASP has some limitations, e.g., WASP does not allow addition of user-defined 5′ tail sequences to ASPs. Additionally, WASP searches for ASP binding sites from only one side (upstream) of a polymorphism site. Furthermore, it appeared that when attempting to target an ASP on an SNP site, WASP places an additional deliberate mismatch at the penultimate base of AS primers. This peculiarity in the primer-design algorithm was proposed by WASP developers as they sought to increase specificity of allele discrimination. However, using ASPs with deliberate mismatches in the 3′ terminal region is controversial, at least because such mismatches are known to decrease overall PCR efficiency and may lead to complete PCR failure.

**TABLE 4:** Comparison between the KASP tool (in the FastPCR suite) and other AS-PCR programs (web-based).

Feature	WASP	PolyMarker	FastPCR
Web site	https://bioinfo.biotec.or.th/WASP	http://www.polymarker.info	http://primerdigital.com/tools/pcr.html
Platform	Web server	Web server	Java Web Start (Oracle)
Primer-designing algorithm	Primer3	Primer3	FastPCR
Detection limit of SNP/InDel alleles	2	2	2-(4)-any
SNP genotyping	yes	yes	yes
InDel genotyping	no	no	yes
Primer-binding site selection to one side or both sides from the polymorphic site	One side	One side	Both sides
Variable base positioning in ASPs (for SNP targeting) related to the primer’s 3′ terminus	At the first base, an additional deliberate mismatch is introduced at the penultimate base	At the first base	At the first, second, or third base, and automatic selection based on thermodynamic calculations
Uses multiple sequence alignment of alleles as input	no	yes	yes
Allows user-defined 5′ tails in ASPs	no	no	yes
Multiplex reaction design	no	no	yes
Inclusion of commercial (e.g. LCG or Merck KGaA) or custom FRET cassette	no	no	yes
Analysis for primer self-dimers and cross-dimers in all multiplexed primer sets	no	no	yes
*T*_*m*_/dG selection for thermodynamic comparisons	no	no	yes
Probe design (TaqMan, MGB)	no	no	yes
Identification of low- and high-complexity sequences with automatic adjustment of primer positions	no	no	yes
BLAST test	no	yes	no
Ability to automatically adjust algorithm to the sequence complexity of an input template	no	no	yes
Potential for wide range applications beyond genotyping (such as diagnostics, quantitation of alleles)	no	no	yes

In contrast, when targeting SNPs our AS-PCR tool automatically places a variable base at the second (penultimate) position of the primer’s 3′ terminus. The selection of an exact position (of the variable base) is driven by thermodynamic considerations. In our opinion, the latter solution provides greater flexibility in equilibrating thermodynamic parameters for all ASPs targeting existing alleles. In this regard, it was published that 3′-terminal and internal oligonucleotide mismatches differ on the effect on duplex stability. Mismatches at the oligonucleotide’s very 3′ terminus actually stabilize its duplex with a template, whereas internal mismatches can be either stabilizing or destabilizing. A destabilizing effect for internal mismatches occurs when there are unfavourable constraints on the geometry of hydrogen bonding in a DNA duplex (Santalucia and Hicks, 2004). In general, the destabilizing effect is more pronounced for mismatches in the penultimate or third position (at the 3′ terminus) and when A or T is a terminal base. These considerations work in our AS-PCR algorithm in a way that the tool generates various candidate ASPs that carry one intended mismatch at different positions and compares their thermodynamic stability. As a result, the program automatically shifts the 3′ terminus of the ASP to produce the best performing ASP. The best performing ASP does not decrease the efficiency of amplification. In our experience, the described approach increases the specificity of allele detection. Importantly, ASPs designed with our AS-PCR tool can be used in PCR driven by either proofreading or non-proofreading DNA polymerases. In our experience, proofreading DNA polymerases (e.g., brands Phire, Phusion from Thermo Scientific) also produce better results when DNA sources for genotyping are not of the highest quality (direct PCR protocol).

A program capable of designing primers for KASP is PolyMarker (http://www.polymarker.info) (Ramirez-Gonzalez et al., 2015). We used example template sequences published on the PolyMarker website to produce primers using PolyMarker and our AS-PCR tool. However, for two example sequences (Cadenza1697_chr1A_12142209 1A; BA00343846 5A), PolyMarker did not perform the expected KASP analysis, as it identified two copies of the target region in the hexaploid wheat genome. In contrast, our AS-PCR tool does not impose similar restrictions because our tool is not only able to check for repeats but can also position ASPs in non-repeated regions. Thus, our AS-PCR tool is more suitable for work with polyploid genomes. When our AS-PCR tool analysed a template in the example above, the tool found a non-repeated region at one side of a polymorphic site of interest and generated ASPs. With two different example templates (BA00591935 3B; BA00122841 7D), PolyMarker and our AS-PCR design tool generated ASPs of the same sequences. However, the computed common reverse primers were different. Our AS-PCR tool’s output with the primers’ *T*_*m*_ illustrates that this program can design reverse primers with thermodynamic properties very similar to ASPs.

The other differences between these programs (WASP, PolyMarker) favour our AS-PCR design tool, as both programs do not allow for addition of predefined 5′ tails to ASPs and do not check primer thermodynamic compatibility if the ASPs have 5′ tails. PolyMarker searches for primer-binding sites at only one side of a polymorphic site, whereas our tool searches in both directions.

It should be mentioned that the primer-design algorithm in software from other developers (WASP and PolyMarker) is significantly different from ours. This difference becomes evident when a sequence surrounding a polymorphic site has low (linguistic) complexity, as in cases of perfect or imperfect microsatellites (simple sequence repeats, SSRs) or G-rich sequences capable of G/C-quadruplex formation. The algorithm in software from other groups ignores the linguistic complexity and sometimes target primers on sequences with low linguistic complexity. During PCR, such primers will generate unexpected amplifications and may hamper identification of specific alleles (Kalendar, 2022).

An important condition for successful PCR is that all primers in one multiplex reaction must have similar thermodynamic properties (*T*_*m*_, dG) and be compatible in terms of possible primer-dimers. Our AS-PCR tool is arguably the most sophisticated instrument in these regards, as the tool automatically checks the compatibility of all primers in a multiplex design.

Thus, the main differences between these programs (WASP, PolyMarker) and our AS-PCR tool are that probe sequences are designed in both directions and both SNPs and InDels may be targeted. Our tool automatically places a variable base at the second (penultimate) position of the primer’s 3′ terminus or to any other position in manual or automatic mode. In addition, our tool allows discrimination of up to four-allelic variants at a single SNP site. Finally, our tool allows design of custom FRET cassette reporter systems for fluorescence-based assays.

### Allele-specific qPCR and AS-PCR Tool Validation

We propose a modification to the KASP method to an improved and universal ‘Allele-specific qPCR’ (ASQ) for designing target-specific primers for KASP genotyping assays. When compared with KASP, this proposed ASQ method also contains two separate components: an allele-specific part (two or more AS primers targeting the SNP/InDel with identity in the penultimate 3′ nucleotide and specific 5′ tags) and a universal part. There are two or more universal probes (UP-1-4) with corresponding tags and different fluorescent dyes at the 5′ end and a single common UP with a quencher at the 3′ end (Uni-Q) (Table 3). A single common UP Uni-Q at the 3′ terminus carries a universal 3′ quencher (Eclipse with quenching range 390–625 nm; Black Hole Quencher [BHQ1] with quenching range 450–580 nm, Black Hole Quencher [BHQ2] with quenching range 520–650 nm, or Tide Quencher 3 [TQ3] with quenching range 510–620 nm) that effectively quenches a broad range of fluorescent dyes for most fluorescently labelled UPs. The identical sequence of the 13-bp tag-fragment in each UP fully complements those in the Uni-Q. Differences in the tail sequences are determined by short barcode 6-nt sequences that are not part of the universal tag sequence and are located between it and ASP sequence (Table 3). The proposed modified KASP method based on FRET is suitable for single- or multiplex applications and can be used in various approaches for SNP/InDel genotyping (Figure 1). During thermal cycling, the relevant ASP binds to a template and elongates, thus attaching its tail sequence to a newly synthesized strand. The complement of the allele-specific tail sequence is generated during subsequent rounds of PCR, enabling the dye-containing component in a FRET cassette to bind to DNA. Through binding, the dye is no longer quenched and emits fluorescence. Separating of the reactions into template-specific and not template-specific parts allows screening of multiple templates in one run. ASQ protocols use FRET master mixes for generating fluorescent signals with different templates in development of various genotyping assays.

## Conclusion

The wide use of sequencing has shown that sequence variations (SNPs and InDels) between individuals of a given species is very common; for example, millions of SNP differences are found between two wheat varieties. It is not always necessary to have access to all this information for comparative studies. For many tasks, a limited number of polymorphic DNA sites is sufficient, and the use of high-density microarrays and NGS is unjustified and excessively costly. Improved AS-PCR techniques (e.g., KASP-related technology) are entirely appropriate in such situations. A limitation of the originally described KASP method is that only two alternative alleles at any specific site can be detected because of the use of individual quencher oligos for each ASP at a FRET cassette. However, planning KASP for the detection of three or four alleles at the same site and for allele identification at different polymorphic sites is possible in one multiplexed reaction. The latter possibility is valuable for those who require multiplexing to decrease sample number and increase throughput. Using a four-plexed fluorescent KASP assay, output during SNP genotyping will be doubled without additional labour or a significant increase in costs.

Here we describe a convenient alternative to originally described fluorescent reporters (FRET cassettes) that employs one universal quencher oligonucleotide. The quencher oligonucleotide is complementary to the UP 5′ tails. The quencher oligonucleotide is also complementary to the 5′ proximal sequence in the fluorescent probes. The quencher oligonucleotide at the 3′ terminus carries a universal quencher that effectively quenches a broad group of fluorescent dyes (such as Eclipse, BHQ1 or BHQ2 or Tide Quencher 2/3). Any of the known non-fluorescent dark quenchers can potentially be used (Fiandaca et al., 2001; Li et al., 2006), as this quenching occurs in non-dual-labelled probes through non-FRET (static) quenching mechanisms (Johansson et al., 2002; Marras et al., 2002) in situations where the dyes are held close together through hybridization. Static quenching occurs through the formation of a ground-state complex and can be important in dual-labelled “linear” probes (Parkhurst and Parkhurst, 1995; Bernacchi and Mely, 2001). Efficient quenching can be obtained via static quenching without the use of molecular beacon stem-loop structures. The oligonucleotide presumably acts as a tether, effectively increasing the relative fluorophore quencher concentration, promoting heterodimer formation.

Differences in the tail UP sequences are determined by a short barcode 6-nt sequence that is not part of the universal tail sequence. The improved KASP makes AS-PCR-based genotyping into a flexible, easy-to-plan, high-throughput, and cost-effective technology. Modern qPCR machines are emerging that allow simultaneous use of four or more optical channels to detect fluorescent signals. Such equipment is poised to be utilized with the improved KASP as it is possible to genotype more SNPs or include more-than-two-variant SNPs.

In this work, we describe a tool working in the FastPCR environment for developing PCR-based genotyping assays. The tool helps design assays for detection of SNPs and InDels. The FastPCR software was originally created with the intention to allow great flexibility when designing PCR assays. Now with KASP capabilities, FastPCR allows for even more sophisticated genotyping assays, which translates into a higher overall success rate and provides the unique ability to genotype mix-type SNP/InDel polymorphisms. Increasing the reaction specificity and discriminative power of an assay, the ASPs are positioned automatically such that the penultimate base at the primer’s 3′ end is placed over the variable base. This feature of the FastPCR algorithm permits the use of proofreading enzymes, which is necessary when working with low-quality DNA templates and with a direct PCR assay. FastPCR expands detection options as it makes possible detection of all four variants at a single SNP site and any form of InDel. Theoretically, it also allows genotyping of several different SNP targets in a multiplexed reaction and genotyping mix-type polymorphisms. This contrasts with the limited ability of standard KASP assays to detect only two variants at a SNP site. FastPCR generates primers to target a polymorphic site from either strand. Custom FRET cassette reporter systems may be devised, and a universal FRET cassette system from LGC Biosearch Technologies may be used in planning the assays.

The AS-PCR design tool may be used to plan high-throughput screening assays to rapidly identify mutant strains of microorganisms, particular genetic lines of higher organisms, or cell lines, such as those produced with genome editing using Transcription Activator-Like Effector Nucleases (TALEN) or Clustered Regularly Interspaced Short Palindromic Repeats/CRISPR-associated protein (CRISPR/Cas) (Lee et al., 2016; Koonin et al., 2017; Shmakov et al., 2017). An important prospective use of our AS-PCR design tool is rapid testing for specific variations in pathogen genomes. For example, the four-way genotyping assay may provide information on genetically encoded drug resistance or the presence of pathogenicity determinants in a clinical isolate. It may also be possible to rapidly design and deploy genotyping assays for epidemiological studies of circulating pathogens. Another entity presented in this manuscript is a KASP modification for quantitative measurement of the presence of allelic variants. The ASQ method is thus a highly multiplexed alternative to traditional quantitative PCR.

## Data Availability

The original contributions presented in the study are included in the article/Supplementary Material, further inquiries can be directed to the corresponding author.

## Abbreviations

ASP, allele-specific primer; AS-PCR, allele-specific PCR; ASQ, allele-specific qPCR; dG, Gibbs free energy; FRET, fluorescence resonant energy transfer; InDel, insertion-deletion polymorphism; KASP, kompetitive allele-specific PCR; SNP, single-nucleotide polymorphism; Uni-Q, universal quencher probe; UP, fluorescently-labeled Universal probe.
