# Supplementary material for: Designing Allele-Specific Competitive-Extension PCR-Based Assays for High-Throughput Genotyping and Gene Characterization
Source: Front Mol Biosci. 2022 Mar 1;9:773956. doi: 10.3389/fmolb.2022.773956 (PMC8921500; doi:10.3389/fmolb.2022.773956)
Supplement: Supplementary file 1 [file DataSheet1.PDF]

Supplemental Material

Designing allele-specific competitive-extension PCR-based assays for high-throughput genotyping and gene characterization

Ruslan Kalendar [1,2,*], Alexandr V. Shustov [3], Ilyas Akhmetollayev [3], Ulykbek Kairov [2]

[1] Institute of Biotechnology HiLIFE, University of Helsinki, P.O. Box 65, FI-00014 Helsinki, Finland

[2] National Laboratory Astana, Nazarbayev University, 010000, Nur-Sultan, Kazakhstan

[3] National Center for Biotechnology, Korgalzhin hwy 13/5, 010000, Nur-Sultan, Kazakhstan

***Correspondence**:

Ruslan Kalendar, ruslan.kalendar@helsinki.fi, phone: +358504483505

R.K.: ruslan.kalendar@helsinki.fi ORCID 0000-0003-3986-2460

A.V.S.: shustov@biocenter.kz ORCID 0000-0001-9880-9382

I.A.: iliyas@mail.ru ORCID 0000-0002-6219-4002

U.K.: ulykbek.kairov@nu.edu.kz ORCID 0000-0001-8511-8064

Running Head: Multiplex genotyping assays design tool

Keywords: single nucleotide polymorphism; insertion-deletion polymorphism; genotyping system; diagnostic system; polymerase chain reaction-based markers; genotyping assay design software

Abbreviations:

ASP (Allele-Specific Primer)

AS-PCR (Allele-specific PCR)

ASQ (Allele-Specific qPCR)

dG (Gibbs free energy)

FRET (Fluorescence Resonant Energy Transfer)

InDel (insertion-deletion polymorphism)

KASP (Kompetitive Allele Specific PCR)

SNP (Single-Nucleotide Polymorphism)

Uni-Q (Universal Quencher probe)

UP (fluorescently-labelled Universal probe)

Supplemental Data 1.

This paragraph presents examples of input information into a FastPCR program. The input contains nucleotide sequences of allelic variants which are used to compute primers for Kompetitive Allele Specific PCR (KASP) or Allele-Specific Quantitative PCR (ASQ). In this example, allele-specific PCR is being designed to genotype SNP alleles. The SNP of interest must be enclosed in [square brackets] and can be formatted as **[First allele/Second allele/Third allele/Fourth allele]** or [**IUPAC code**] or [**Target Nucleotide**]:

Example 1. Formatting sequences for SNP:
>1
tcatattccagtttgggcgagttttaagataggtccgg **[C/G]** acagtctttgcggcgccaacgcgtctttctccagcagacagtccccggactgc
>2
tcatattccagtttgggcgagttttaagataggtccgg **[S]** acagtctttgcggcgccaacgcgtctttctccagcagacagtccccggactgc
>3
tcatattccagtttgggcgagttttaagataggtccgg **[C]** acagtctttgcggcgccaacgcgtctttctccagcagacagtccccggactgc

Allele-specific PCR (AS-PCR) assays can be designed for discrimination of insertions/deletions (InDels) polymorphisms. The program imposes no size limit on length difference for InDels alleles, provided that all alleles can be aligned, and the alignment has a sufficient length of overlapping to target primers. To submit variants with a large InDels polymorphism, at least 12 bp before the start of the non-identical region should be present in all sequences. Two, three or four allelic variants may be included for analysis to compute ASPs and UP. Only differences between the variants must be shown within the brackets. One Allele-Specific Primer (ASP) will be computed for each of the input allelic variants. Also, one common primer (Universal Primer, UP) will be computed which targets a conserved region in all sequences. Input sequences should be in a FASTA format with differences between alleles placed within square brackets: **[allele1/allele2/allele3/allele4]**.

Example 2. Formatting sequences for InDels:
>1
tcatattccagtttgggcgagttttaagataggtccgg **[AG/-]** acagtctttgcggcgccaacgcgtctttctccagcagacagtccccggactgc
>2
tcatattccagtttgggcgagttttaagataggtccgg **[AG/TT]** acagtctttgcggcgccaacgcgtctttctccagcagacagtccccggactgc

To instruct the software to design an AS-PCR assay, a user needs to put the "***-ASPCRn***" command in the FASTA header of a specific sequence, or globally within curved brackets **{ }**. The command has an option to request the software to position the first, second or third base at the ASP's 3'-terminus over the SNP site. For this purpose, "n" in the command string must be substituted with a number. A default is ***-ASPCR2*** (which means that a variable base is in the penultimate position in the ASP's 3'-terminus).

If user do not specify the number: ***-ASPCR***, in this case the program will automatically determine the optimal length for effective discrimination of single nucleotide polymorphisms.

If one needs to add user-defined tails to ASP's 5'-termini, a user can do this by defining a specific 5'-tail for any ASP. For this purpose, a tail sequence must be included in the FASTA header, after the "-p5e" command and individual tail-sequences must be separated by the slash "/".

For example, to utilize universal FRET-cassettes from LGC Biosearch Technologies (which are FAM-GAAGGTGACCAAGTTCATGCT and HEX-GAAGGTCGGAGTCAACGGATT), input data should contain the following FASTA header:

>1 ***-ASPCR*** -p5e[gaaggtgaccaagttcatgct/gaaggtcggagtcaacggatt]
tcatattccagtttgggcgagttttaagataggtccgg **[AG/TT]** acagtctttgcggcgccaacgcgtctttctccagcagacagtccccggactgc

Example 3. It is also possible to specify user-defined 5'-tails for three or four ASPs:

>1 ***-ASPCR*** -p5e[gaaggtgaccaagttcatgct/gaaggtcggagtcaacggatt/gaaggtcggagtcaacggacc]
tcatattccagtttgggcgagttttaagataggtccgg [ATAT/CC/A] acagtctttgcggcgccaacgcgtctttctccagcagacagtccccggactgc

Comment: in this example, the FASTA header contains commands after the ">" sign, and then goes a list of tail-sequences for three ASPs. Starting from the next line, there is a target sequence representing all three alleles. Differences between the alleles (e.g. the polymorphism of interest) are enclosed in square brackets.
There is no need to explicitly specify tail-sequences for all ASPs. The tool is sufficiently flexible to add specified tails for only desired ASPs.

AS-PCR example 4: Four-plex fluorescent ASQ assay:
In the ASQ method, a PCR contains ASPs (four ASP for four alleles in this example), common universal primer (UP), and a detector system. The detector system includes Universal Probes (UProbe, there is the same number of UProbes as the number of ASPs, four in this example) and a universal quencher oligonucleotide (Uni-Q). To design four-plex ASQ, the FastPCR input is:

>**example4 *-ASPCR2*** -Ln18-28 -Tm53-56 -
p5e[ccagctgaacggtGCTCAC/ccagctgaacggtTGCGAC/ccagctgaacggtGTGCGA/ccagctgaacggtGCGTCA]
CTTAGATCGACAGGTCTAAGAGCTGAAGAGCTAGCTATTAAAGTCGAGC**[n]**AGCTGCTAGACGTCGCAGTCGACACAGCTAGCC
TAGGACAAAGTCTCGTG

Target sequence contains an SNP polymorphism with 4 states: **[n]**;

AS-PCR command: ***-ASPCR2*** for located SNP at the penultimate base in 3'-end of each allele-specific primer (ASP); command: -Ln18-28 -Tm53-56 - for determination of length of primers (between 18 and 28 nt) and Tm for primers between 53° to 56°C; command: -p5e[ccagctgaacggtGCTCAC/ccagctgaacggtTGCGAC/ccagctgaacggtGTGCGA/ccagctgaacggtGCGTCA] - defines a 5'-tail for each allelic variant of ASPs. The sequence of fluorescently-labelled universal probe (UP) with a single universal quencher probe (Uni-Q) is presented in **Table 2**.

The FastPCR output for Example 4:

PrimerID	Sequence (5'-3')	nt	Tm (°C)	dG (kcal/mol)	Tm 3'end (°C)	GC (%)	LC (%)	PQ (%)	Fragment Size (bp)/ Tm (°C)	Topt (°C)
1:1F_1_30-49_ASP1	CCAGCTGAACGGTGCTCACtagctattaaagtcgagcGA	20	55.9	-22.8	44.2	40.0	82	77		
1:1F_1_30-49_ASP2	CCAGCTGAACGGTTGCGACtagctattaaagtcgagcCA	20	55.9	-22.7	43.9	40.0	89	89		
1:1F_1_28-49_ASP3	CCAGCTGAACGGTGTGCGAgctagctattaaagtcgagcAA	22	58.3	-25.4	40.5	40.9	82	82		
1:1F_1_28-49_ASP4	CCAGCTGAACGGTGCGTCAgctagctattaaagtcgagcTA	22	57.3	-24.8	38.5	40.9	75	56		
1:R_80-97	gagactttgtcctaggct	18	55.9	-21.1	41.6	50.0	89	89	68/76	60
1:1F_1_30-49_ASP1	CCAGCTGAACGGTGCTCACtagctattaaagtcgagcGA	20	55.9	-22.8	44.2	40.0	82	77		
1:1F_1_30-49_ASP2	CCAGCTGAACGGTTGCGACtagctattaaagtcgagcCA	20	55.9	-22.7	43.9	40.0	89	89		
1:1F_1_28-49_ASP3	CCAGCTGAACGGTGTGCGAgctagctattaaagtcgagcAA	22	58.3	-25.4	40.5	40.9	82	82		
1:1F_1_28-49_ASP4	CCAGCTGAACGGTGCGTCAgctagctattaaagtcgagcTA	22	57.3	-24.8	38.5	40.9	75	56		
1:R_77-94	actttgtcctaggctagc	18	56.3	-21.3	42.7	50.0	81	76	65/76	60
1:1F_1_31-50_ASP1	CCAGCTGAACGGTGCTCACctgcgacgtctagcagctGG	20	66.3	-27.7	44.2	65.0	84	79		
1:1F_1_31-50_ASP2	CCAGCTGAACGGTTGCGACctgcgacgtctagcagctCG	20	66.2	-27.9	44.5	65.0	84	84		
1:1F_1_28-50_ASP3	CCAGCTGAACGGTGTGCGAcgactgcgacgtctagcagctAG	23	67.6	-31.2	38.8	60.9	76	53		
1:1F_1_30-50_ASP4	CCAGCTGAACGGTGCGTCAactgcgacgtctagcagctTG	21	65.6	-28.3	40.8	57.1	87	87		
1:R_74-92	gacaggtctaagagctgaa	19	56.3	-22.0	35.7	47.4	89	89	62/75	60

Examples of FastPCR windows with analysis setup

A user selects the checkbox with a task required. The program will only perform the selected task. Depending on which task the user selects, pressing **F5** will execute the current task. The user either presses **F5** or clicks on the toolbar to execute the current task (press **Run**). Once the task is complete, a Result window will be populated with data. **Figure S1** shows a sample result visualization window.

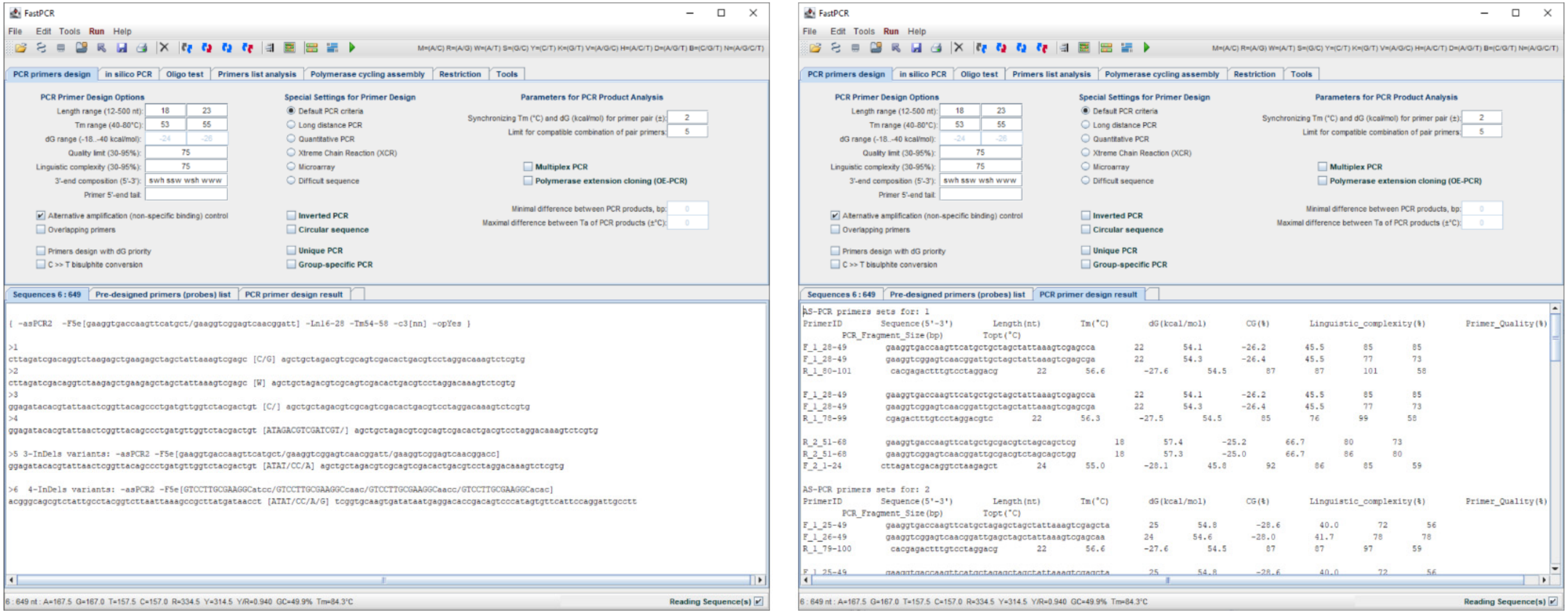

Figure S1. The figure on the left shows 6 examples for performing an AS-PCR task. The examples are shown here demonstrate the design capabilities of the AS-PCR set both single nucleotide polymorphisms (SNPs) and insertion-deletions (InDels) may be targeted. In addition, specific tags set can be selected for a specific polymorphism (target). The result of the AS-PCR set design is shown in the Result report (right picture).

PCR primer design options

The PCR Primer Options box allows users to designate basic parameters for the PCR reaction and the primers that are generated.

For individual PCR primer design options for each sequence, a user can type commands in a header of the sequence. Typically, the user does not need to use commands to manage PCR-primer design; these commands are optional and only needed for advanced users:

-ln21-25 -tm54-56 -3tm25-40 -q75 -lc75 -npr5000 -c5[nn] -c3[swh ssw wsh sww www] -dmr=1 (20-5000),

where '-ln21-25' determines the range of primer length (21-25 bases);

'-tm54-56' determines the range of primer Tm (54-56°C);

'-3tm25-40' determines the range of primer Tm at 3'end (25-40°C);

'-npr5000' shows the limit for maximal primer amount designed for each target (5000);

'-dmr=1' sensitivity for primer-dimer detection, default value is 1 (as the strictest criterion), the higher the value, the lower the detection sensitivity, if the value is 0, the program will not assess for the presence of primer-dimers, the optimal value of this parameter is 2, as the most realistic;

(20-5000) boundaries of the size of PCR amplicons, the exact size is allowed, in this case, the size of the PCR amplicon will be in the range of 20 to 5000 base pairs;

'-c5[nn]' primer have no specific sequence pattern for 5' ends;

'-c3[swh ssw wsh sww www]' specifies for primers 3'ends with these patterns with three bases per pattern.

Supplemental Data 2.

PolyMarker is an automated bioinformatics pipeline for SNP assay development which increases a probability of generating homologue-specific assays for polyploid species. PolyMarker generates a multiple alignment between a target SNP sequence and a selected reference genome. It then generates a mask with informative polymorphisms positions which are highlighted for inspection.
http://www.polymarker.info/

```
Input sequences examples:
Cadenza1697.chr1A.12142209,1A,acaactttcaaaaaataacacacccacaacctaaacacccttaaatatcaacttctgaggggaggcggcaactagtatcaaaaattcaaaatgtggttc[G/C]cacaactagc
gttgccgagacaaaaagaaccagcacttccactgctccatgaattcaagaaacagaccgatggcatgtataagaacagacgtgccga
BA00591935,3B,gcacatcggagagttcgttggaagagcctagcaccgaggaattccaggaggaagatgtctctgatggagattcagactcgaacgacgagagcaaggggcc[G/A]gaagtaaagcttttcatcagcggagt
agttcataataaagaggaggctggagcaaaatcttatgttcgagttcccgctgaaataaataacctggaaaggg
BA00343846,5A,ccaccccctcctcttccccatgcagctacaactgaggcaatgctgccgtctgccttctggagttccccgccttcgatctcgagcacgccatccaggacggc[G/A]ttggtatcgaccgccaacctcaatct
ggatccggttgtggctaatgtacagactaattcccacacaaaacattcattaagttagcattgtctctttttgg
BA00122841,7D,ttcccaccacacgcttcagcagttccttggctcgcgatgaccccgactcatcatggcagaactgggtccggaggcccccatcaagacccttgtaaatgta[A/G]aagtacactgccagttgttcgctgcc
gctgttggtggcatggatgagaaggccaaaggggccaagcgcgccgcgttcagtggcaccaccacttgtactgc
```

PolyMarker Output result:

Target			ASP1	ASP2	Reverse
Cadenza1697_chr1A_12142209	1A	1	none	none	none
BA00591935	3B	2	cgctgatgaaaagctttacttc**C**	cgctgatgaaaagctttacttc**T**	gttggaagagcctagcacc
BA00343846	5A	0	none	none	none
BA00122841	7D	3	cgaacaactggcagtgtactt**T**	cgaacaactggcagtgtactt**C**	ttggctcgcgatgacccc

Example. FastPCR program computes primers for AS-PCR using input sequences from examples at the PolyMarker website. Sequences were prepared in the FASTA format. FASTA headers contain commands to instruct FastPCR to make KASP primers.

```
{ -asPCR1 -Ln18-28 -Tm53-56 }
>Cadenza1697.chr1A
acaacttttcaaaaaataacacacccacaacctaaacacccttaaatatcaacttctgaggggaggcggcaactagtatcaaaaattcaaaatgtggttc[G/C]cacaactagcgttgccgagacaaaaagaaccagcacttcc
actgctccatgaattcaagaaacagaccgatggcatgtataagaacagacgtgccga
>BA00591935
gcacatcggagagttcgttggaagagcctagcaccgaggaattccaggaggaagatgtctctgatggagattcagactcgaacgacgagagcaaggggcc[G/A]gaagtaaagcttttcatcagcggagtagttcataataaag
aggaggctggagcaaaatcttatgttcgagttcccgctgaaataaataacctggaaaggg
>BA00343846
ccacccctcctcttccccatgcagctacaactgaggcaatgctgccgtctgccttctggagttccccgccttcgatctcgagcacgccatccaggacggc[G/A]ttggtatcgaccgccaacctcaatctggatccggttgtgg
ctaatgtacagactaattcccacacaaaacattcattaagttagcattgtctctttttgg
>BA00122841
ttcccaccacacgcttcagcagttccttggctcgcgatgaccccgactcatcatggcagaactgggtccggaggcccccatcaagacccttgtaaatgta[A/G]aagtacactgccagttgttcgctgccgctgttggtggcat
ggatgagaaggccaaaggggccaagcgcgccgcgttcagtggcaccaccacttgtactgc
```

FastPCR Output result:

PrimerID	Sequence (5'-3')	nt	Tm (°C)	dG (kcal/mol)	Tm 3'end (°C)	GC (%)	LC (%)	PQ (%)	Fragment Size (bp) / Tm (°C)	Topt (°C)
cadenza1697.chr1a:1F_1_74-100_ASP1	agtatcaaaaattcaaaatgtggttc**G**	27	53.6	-29.5	33.4	29.6	71	56		
cadenza1697.chr1a:1F_1_74-100_ASP2	agtatcaaaaattcaaaatgtggttc**C**	27	53.3	-29.2	32.1	29.6	71	61		
cadenza1697.chr1a:1R_178-199	tcggcacgtctgttcttataca	22	55.4	-27.1	25.0	45.5	88	87	126/77	58
cadenza1697.chr1a:1R_2_102-120_ASP1	tcggcaacgctagttgtg**C**	19	57.9	-26.0	38.9	57.9	92	75		
cadenza1697.chr1a:1R_2_102-120_ASP2	tcggcaacgctagttgtg**G**	19	57.2	-25.6	37.5	57.9	92	77		
cadenza1697.chr1a:1F_32-59	ctaaacacccttaaatatcaacttctga	28	53.4	-29.9	29.4	32.1	80	80	89/75	58
ba00591935:2F_1_82-100_ASP1	cgacgagagcaaggggcc**G**	19	62.5	-27.9	47.4	73.7	70	45		
ba00591935:2F_1_82-100_ASP2	cgacgagagcaaggggcc**A**	19	61.0	-27.3	45.0	68.4	70	43		
ba00591935:2R_170-194	aggttatttatttcagcgggaactc	25	55.2	-28.9	40.9	40.0	86	81	113/77	60
ba00343846:3F_1_82-100_ASP1	cacgccatccaggacggc**G**	19	63.2	-28.3	46.8	73.7	78	68		
ba00343846:3F_1_82-100_ASP2	cacgccatccaggacggc**A**	19	61.6	-27.6	44.5	68.4	78	73		
ba00343846:3R_171-198	aaagagacaatgctaacttaatgaatgt	28	53.6	-30.2	23.8	28.6	78	78	117/78	58

ba00343846:3R_2_102-120_ASP1	gttggcggtcgataccaa**C**	19	55.8	-24.8	32.8	57.9	95	67		
ba00343846:3R_2_102-120_ASP2	gttggcggtcgataccaa**T**	19	54.4	-24.3	31.1	52.6	95	93		
ba00343846:3F_10-30	ctcttccccatgcagctacaa	21	56.2	-26.4	34.9	52.4	87	82	111/84	60
ba00122841:4F_1_76-100_ASP1	cccatcaagacccttgtaaatgta**A**	25	54.8	-28.6	22.8	40.0	84	60		
ba00122841:4F_1_77-100_ASP2	ccatcaagacccttgtaaatgta**G**	24	53.4	-26.9	23.3	41.7	88	67		
ba00122841:4R_109-126	cagcgaacaactggcagt	18	54.9	-23.7	38.4	55.6	81	81	51/71	59
ba00122841:4R_2_102-123_ASP1	cgaacaactggcagtgtactt**T**	22	55.5	-27.0	32.3	45.5	88	87		
ba00122841:4R_2_102-122_ASP2	gaacaactggcagtgtactt**C**	21	53.6	-25.0	33.1	47.6	87	87		
ba00122841:4F_6-23	accacacgcttcagcagt	18	55.8	-24.2	40.9	55.6	78	78	118/82	60

WASP Web-based Allele Specific Primer design tool
https://bioinfo.biotec.or.th/WASP

```
>SNPID 1
CAAGCCGGGCTACGTCCGAGGGTAACAACATGATCAAAACCACAGCAGGAACCACAATAAGGAACAAGACTCAGGTTAAAGCAAACACAGCGACAGCTCCTGCGCCGCATCTCCTGGTTCCAGTGGCGGCACTGAACTCGCGGCA
ATTTGTCCCGCCTCTTTCGCTTCACGGCAGCCAATCGCTTCCGCCAGAGAAAGAAAGGCGCCGAAATGAAACCCGCCTCCGTTCGCCTTCGGAACTGTCGTCACTTCCGTCCTCAGACTTGGAGGGGCGGGGATGAGGAGGGCGG
GGAGGACGACGAGGGCGAAGAGGGTGGGTGAGAGCCCCGGAGCCCGAGCCGAAGGGCGAGCCGCAAACGCTAAGTCGCTGGCCATTGGTGGACATGGCGCAGGCGCGTTTGCTCCGACGGGCCGAATGTTTTGGGGCAGTGTTTT
GAGCGCGGAGACCGCGTGATACTGGATGCGCATGGGCATACCGTGCTCTGCGGCTGCTTGGCGTTGCTTCTTCCTCCAGAAGTGGGCGCTGGGCAGTCACGCAGGGTTTGAACCGGAAGCGGGAGTAGGTAGCTGCGTGGCTAAC
GGAGAAAAGAAGCCGTGGCC[G/A]GGGAGGAGGCGAGAGGAGTCGGGATCTGCGCTGCAGCCACCGCCGCGGTTGATACTACTTTGACCTTCCGAGTGCAGTGGTAGGGGCGCGGAGGCAACGCAGCGGCTTCTGCGCTGGGAA
ATTCAGTCGTGTGCGACCCAGTCTGTCCTCTCCCCAGACCGCCAATCTCATGCACCCCTCCAGAGTGGCCCTTGACTCCTCCCTCTCCTCACTCCATCTTTCCTGGCCTCTCTCCGGGTGCTTAGCGGACTTGGCCAATAACCTC
CTCCTTTTAAACGCCCTGAATTGAACCCTGCCTCCTGCGCATCCTCTTTTTGTGTCACCTTAGGGTTCAGATTTAACTACGCGACTTGACTAGTCATCTTTTGATCTCTCTCTCGTATTTAGTACTTTTAGTCAGCGAGCATTTA
TTGATATTTCAACTTCAGCCTCGCGGTTAAGAGCTTGGGCTCTGGAATCATACGGCTGGAATTGGAATTCTGTCAGTCGTGTGGCCGCTCTCTACTGTCTTGTGAAGATAAGTGAGATAATCTTGACCTGTGGTGAGCACTCGTG
AGCGTTAGCTGCTGTATTTACCAGGTACAGATAAGACAACTACA
```

Oligo 1 :	Pos.	Len.	Tm	GC%	Self Any	Self End
Wildtype Reverse Primer 5': TCCTCTCGCCTCCTCCCTC	619	19	64.29	68.42	2.00	0.00
Mutant Reverse Primer 5': TCCTCTCGCCTCCTCCCTT	619	19	63.66	63.16	2.00	0.00
Common Forward Primer 5': TTGCTTCTTCCTCCAGAAGT	499	20	56.70	45.00	5.00	2.00
Product Size: 121						

Oligo 2 :	Pos.	Len.	Tm	GC%	Self Any	Self End
Wildtype Reverse Primer 5': CCTCTCGCCTCCTCCCTC	618	18	62.43	72.22	2.00	0.00
Mutant Reverse Primer 5': CCTCTCGCCTCCTCCCTT	618	18	61.83	66.67	2.00	0.00
Common Forward Primer 5': CTTCTTCCTCCAGAAGTGG	502	19	54.88	52.63	5.00	2.00
Product Size: 117						

```
>SNPID 2
GACCCTGAAGCTCCAGCGTGAGGCCTGGCATTGAATGAAATATATTTTGTGGGTTTTCAGCTGCTGAAGTCATAGGAATGGATGAGACCAAGAAAACAAAGCTGTTTTTGAGGTATGAGCGGAAGAAGAGATATCAGGAGACTTT
CGAAACAGTCATAACGGAAGTTAATATGATCATTGCTAACATTTGCTGTGTTTCAGGCACTGTAAGCATGTATATGGGTCCTTAAAGGGACTCATAGAGGTAGGTACTAGTATTGTTTTTCCTTTTATCATTGAGAAACTGAGGT
TTGAAGAGATTAGTGAACTTGCTCTAGATTATACAGTTTGTAAGTGGCTGAACCAGGATTTGAACTAATACAATCTGACTACAGAGGCCACACTCCTTAGCACTAGAAAAGAATGGCATGCCAAGGGCAGAGTTATTTCTAGGAA
GATGGGATATAAGCGTCATTGTCAAGTTGTGCAAAGGGGTCAACTTGGTTGAGATCTAAAAAGGAACACTGAATTCGTCAATTCAGAGGCTCGTAGGAAGTAGGAAATCCTTATCTTTTCGTTTGAACCTTGAGTGGAACCTAGA
TATTCTGGATTAATGTATAA[A/G]TGAAGTTCATCTTTATATGGTGACACTCATACTCTAGTTGACTACCTAATAGTTCCTCTGCCTTCTTTCAGTTCCGCCAACATACTGTGTTCTTTAACATTGTAGAACCCTTTGGCTATG
CTAGTCTCCTTCCCTGAAGTGTTGGTCCTCTAGCCCTTTTTTTGATTGGCACCAGTTAGTTCAGATCTTGGCTTTATTATAAACTCCTCTGAGATATATGTTCCCTGACAGTTTATCTAAAATGATTCTCTCTACAGTTATTTTC
TATTACTGTGTTTTTGTTTCCTCAGAGGCATACATCACAATTTGGAATTATGCATTGGTTTATCAATTTACTTGTTTATTGTCACCCTGCTGCCCAGATATGACTTCATGAGGGTAGGATTTGTATCTGTTTAGTTCATTATTTG
TCTAGCTATAAGTAGTAAATATTGTTTGCATCTATCAGTGAATGAGCATCTTCTGTTTATGTAGATAATACTGAACTGTTTTGTTTTCTTCAAAGTAATCATTAAGCTGAAGGTAAGAAAAACAAGTTCAAGGAAGTCACACCAT
GGGGAAAAAAAAGTCAAGTTTAGGGCTGCCTCTTCCGGGAAGTGT
```

No result for this SNP

```
>SNPID 3
CTGTTTTCTATTCATCTTTCATGTCTCAATTTAAATGTCATGTTTCTTTGGTCTCAGAGTAATAAAAAGTAAATATACTTCCCCCATCTCCCGCCCCGCAGAGCCTTCCTCTGTTGCCCAGGCTGGAGTACAGTGGCTCGATCTT
GGCTCACTGAAAGCTCTGCCTCCCAGATTCAAGCGATTCTCCTGCCTCAGCCTCCTGAGTAGCTGGGATTACAGGCGCGCGCCACCAAGCCCGGCTAATTTTTGTATTTTTAGTAGAGACAGGGTTTCACCATGTTGGTCAGGCT
GGTCGAACTCCTGACCTTGTGATCCTCCCACCTTGGCCTCCCAAAGTGCTGGGATTACAGGCGTGAGCCACTGCGTCCAGTGTAAATTATACTTTTATTTTAATCCTGCTACTACTGCAAGCAAGGCAAACATTTTTGTGTTACA
GCATTACTTGTATAGATTTTAAGAAAATCTCATTTTAAATACGGAAATGTTAAGAAAAATTATTGTGCCTTTGACCAGAATGTGCCTCTAATTGTACAGTTAAATCTAACTATAAATACTGCAGTATAAAATAATTATATACACA
TTTTTTCACACCTCTTTCTC[T/C]CTATATATGCATATATACATATACATATATATACCTATATGTATTTTTTTTACAGACAGTGATGTGTGTTCTGAAATTGTGAACCATGAGTCTAGTACTTAATGATCTGCTTATCTGCTG
CCGTCAACTAGAACATGATAGAGCTACAGAACGAAAGGTAGTAAATTACTTAAATTCAATTTTTCCTTGAAATAAGTGTGATTAGTAACCCATTATTATTTCCTTTTTATTTTCAGAAAGAAGTTGAGAAATTTAAGCGCCTGAT
TCGAGATCCTGAAACAATTAAACATCTAGATCGGCATTCAGATTCCAAACAAGGAAAATATTTGAATTGGGATGCTGTTTTTAGGTATTCTATTCAAATTTATTTTACTGTCTTTATTTTTCTCTTTCATATTTATTTCTGTTGT
GATATTACTTTTGTGTGTAAGTCTTAACATTTATCTTTGCTTCCTATATATCATTATGCCTTGCATATGAATTTGGCATTTAATATTTATCCAAAACATAATTTTTAAAGGTTGTTCATATAGAAACTTAAAAATTATAAATTAT
TTCTTCAATAAAATGTTTTAGACATATCTCACTCAAAATTGAGAG
```

```
Oligo 1 :                                                        Pos.    Len.     Tm       GC%    Self Any   Self End
Wildtype Forward Primer 5': TTTCACACCTCTTTCTAT                   584     18      44.60     33.33     2.00      2.00
Mutant Forward Primer 5': TTTCACACCTCTTTCTAC                     584     18      44.98     38.89     2.00      0.00
Common Reverse Primer 5': ATCATGTTCTAGTTGACGGC                   740     20      55.23     45.00     4.00      2.00
Product Size: 157
```

Example. FastPCR program computes primers for AS-PCR using input sequences from the WASP website.
Sequences were prepared in the FASTA format. FASTA headers contain commands to instruct FastPCR to make KASP primers.

```
{ -aspcr2 -Ln18-32 -Tm51-54 (100-200)}
>SNPID1
CAAGCCGGGCTACGTCCGAGGGTAACAACATGATCAAAACCACAGCAGGAACCACAATAAGGAACAAGACTCAGGTTAAAGCAAACACAGCGACAGCTCCTGCGCCGCATCTCCTGGTTCCAGTGGCGGCACTGAACTCGCGGCA
ATTTGTCCCGCCTCTTTCGCTTCACGGCAGCCAATCGCTTCCGCCAGAGAAAGAAAGGCGCCGAAATGAAACCCGCCTCCGTTCGCCTTCGGAACTGTCGTCACTTCCGTCCTCAGACTTGGAGGGGCGGGGATGAGGAGGGCGG
GGAGGACGACGAGGGCGAAGAGGGTGGGTGAGAGCCCCGGAGCCCGAGCCGAAGGGCGAGCCGCAAACGCTAAGTCGCTGGCCATTGGTGGACATGGCGCAGGCGCGTTTGCTCCGACGGGCCGAATGTTTTGGGGCAGTGTTTT
GAGCGCGGAGACCGCGTGATACTGGATGCGCATGGGCATACCGTGCTCTGCGGCTGCTTGGCGTTGCTTCTTCCTCCAGAAGTGGGCGCTGGGCAGTCACGCAGGGTTTGAACCGGAAGCGGGAGTAGGTAGCTGCGTGGCTAAC
GGAGAAAAGAAGCCGTGGCC[G/A]CGGGAGGAGGCGAGAGGAGTCGGGATCTGCGCTGCAGCCACCGCCGCGGTTGATACTACTTTGACCTTCCGAGTGCAGTGGTAGGGGCGCGGAGGCAACGCAGCGGCTTCTGCGCTGGGA
AATTCAGTCGTGTGCGACCCAGTCTGTCCTCTCCCCAGACCGCCAATCTCATGCACCCCTCCAGAGTGGCCCTTGACTCCTCCCTCTCCTCACTCCATCTTTCCTGGCCTCTCTCCGGGTGCTTAGCGGACTTGGCCAATAACCT
CCTCCTTTTAAACGCCCTGAATTGAACCCTGCCTCCTGCGCATCCTCTTTTTGTGTCACCTTAGGGTTCAGATTTAACTACGCGACTTGACTAGTCATCTTTTGATCTCTCTCTCGTATTTAGTACTTTTAGTCAGCGAGCATTT
ATTGATATTTCAACTTCAGCCTCGCGGTTAAGAGCTTGGGCTCTGGAATCATACGGCTGGAATTGGAATTCTGTCAGTCGTGTGGCCGCTCTCTACTGTCTTGTGAAGATAAGTGAGATAATCTTGACCTGTGGTGAGCACTCGT
GAGCGTTAGCTGCTGTATTTACCAGGTACAGATAAGACAACTACA
>SNPID2
GACCCTGAAGCTCCAGCGTGAGGCCTGGCATTGAATGAAATATATTTTGTGGGTTTTCAGCTGCTGAAGTCATAGGAATGGATGAGACCAAGAAAACAAAGCTGTTTTTGAGGTATGAGCGGAAGAAGAGATATCAGGAGACTTT
CGAAACAGTCATAACGGAAGTTAATATGATCATTGCTAACATTTGCTGTGTTTCAGGCACTGTAAGCATGTATATGGGTCCTTAAAGGGACTCATAGAGGTAGGTACTAGTATTGTTTTTCCTTTTATCATTGAGAAACTGAGGT
TTGAAGAGATTAGTGAACTTGCTCTAGATTATACAGTTTGTAAGTGGCTGAACCAGGATTTGAACTAATACAATCTGACTACAGAGGCCACACTCCTTAGCACTAGAAAAGAATGGCATGCCAAGGGCAGAGTTATTTCTAGGAA
GATGGGATATAAGCGTCATTGTCAAGTTGTGCAAAGGGGTCAACTTGGTTGAGATCTAAAAAGGAACACTGAATTCGTCAATTCAGAGGCTCGTAGGAAGTAGGAAATCCTTATCTTTTCGTTTGAACCTTGAGTGGAACCTAGA
TATTCTGGATTAATGTATAA[A/G]TGAAGTTCATCTTTATATGGTGACACTCATACTCTAGTTGACTACCTAATAGTTCCTCTGCCTTCTTTCAGTTCCGCCAACATACTGTGTTCTTTAACATTGTAGAACCCTTTGGCTATG
CTAGTCTCCTTCCCTGAAGTGTTGGTCCTCTAGCCCTTTTTTTGATTGGCACCAGTTAGTTCAGATCTTGGCTTTATTATAAACTCCTCTGAGATATATGTTCCCTGACAGTTTATCTAAAATGATTCTCTCTACAGTTATTTTC
TATTACTGTGTTTTTGTTTCCTCAGAGGCATACATCACAATTTGGAATTATGCATTGGTTTATCAATTTACTTGTTTATTGTCACCCTGCTGCCCAGATATGACTTCATGAGGGTAGGATTTGTATCTGTTTAGTTCATTATTTG
TCTAGCTATAAGTAGTAAATATTGTTTGCATCTATCAGTGAATGAGCATCTTCTGTTTATGTAGATAATACTGAACTGTTTTGTTTTCTTCAAAGTAATCATTAAGCTGAAGGTAAGAAAAACAAGTTCAAGGAAGTCACACCAT
GGGGAAAAAAAAGTCAAGTTTAGGGCTGCCTCTTCCGGGAAGTGT
>SNPID3
CTGTTTTCTATTCATCTTTCATGTCTCAATTTAAATGTCATGTTTCTTTGGTCTCAGAGTAATAAAAAGTAAATATACTTCCCCCATCTCCCGCCCCGCAGAGCCTTCCTCTGTTGCCCAGGCTGGAGTACAGTGGCTCGATCTT
GGCTCACTGAAAGCTCTGCCTCCCAGATTCAAGCGATTCTCCTGCCTCAGCCTCCTGAGTAGCTGGGATTACAGGCGCGCGCCACCAAGCCCGGCTAATTTTTGTATTTTTAGTAGAGACAGGGTTTCACCATGTTGGTCAGGCT
GGTCGAACTCCTGACCTTGTGATCCTCCCACCTTGGCCTCCCAAAGTGCTGGGATTACAGGCGTGAGCCACTGCGTCCAGTGTAAATTATACTTTTATTTTAATCCTGCTACTACTGCAAGCAAGGCAAACATTTTTGTGTTACA
GCATTACTTGTATAGATTTTAAGAAAATCTCATTTTAAATACGGAAATGTTAAGAAAAATTATTGTGCCTTTGACCAGAATGTGCCTCTAATTGTACAGTTAAATCTAACTATAAATACTGCAGTATAAAATAATTATATACACA
TTTTTTCACACCTCTTTCTC[T/C]CTATATATGCATATATACATATACATATATATACCTATATGTATTTTTTTTACAGACAGTGATGTGTGTTCTGAAATTGTGAACCATGAGTCTAGTACTTAATGATCTGCTTATCTGCTG
CCGTCAACTAGAACATGATAGAGCTACAGAACGAAAGGTAGTAAATTACTTAAATTCAATTTTTCCTTGAAATAAGTGTGATTAGTAACCCATTATTATTTCCTTTTTATTTTCAGAAAGAAGTTGAGAAATTTAAGCGCCTGAT
```

TCGAGATCCTGAAACAATTAAACATCTAGATCGGCATTCAGATTCCAAACAAGGAAAATATTTGAATTGGGATGCTGTTTTTAGGTATTCTATTCAAATTTATTTTACTGTCTTTATTTTTCTCTTTCATATTTATTTCTGTTGT
GATATTACTTTTGTGTGTAAGTCTTAACATTTATCTTTGCTTCCTATATATCATTATGCCTTGCATATGAATTTGGCATTTAATATTTATCCAAAACATAATTTTTAAAGGTTGTTCATATAGAAACTTAAAAATTATAAATTAT
TTCTTCAATAAAATGTTTTAGACATATCTCACTCAAAATTGAGAG

FastPCR Output result:

No result for this snpid1, because the portion with a high content of G/C pairs

PrimerID	Sequence (5'-3')	nt	Tm (°C)	dG (kcal/mol)	Tm 3'end (°C)	GC (%)	LC (%)	PQ (%)	Fragment Size (bp) / Tm (°C)	Topt (°C)
**snpid2**:2F_1_570-600_ASP1	gaacctagatattctggattaatgtataa**A**t	31	51.6	-30.7	17.1	25.8	82	53		
snpid2:2F_1_572-600_ASP2	acctagatattctggattaatgtataa**G**t	29	51.4	-29.3	20.3	27.6	83	60		
snpid2:2R_739-757	ggctagaggaccaacactt	19	52.8	-23.3	35.3	52.6	86	80	188/78	57
snpid2:2R_2_603-628_ASP1	gtcaccatataaagatgaacttca**T**t	26	51.5	-27.4	26.8	30.8	86	77		
snpid2:2R_2_603-627_ASP2	tcaccatataaagatgaacttca**C**t	25	51.6	-26.8	29.9	32.0	81	81		
snpid2:2F_435-457	agatgggatataagcgtcattgt	23	53.2	-26.5	36.1	39.1	88	88	194/77	57
**snpid3**:3F_1_578-600_ASP1	attttttcacacctctttctc**T**c	23	51.0	-25.1	30.2	34.8	51	38		
snpid3:3F_1_581-600_ASP2	ttttcacacctctttctc**C**c	20	51.4	-23.3	34.0	45.0	55	39		
snpid3:3R_745-769	aatttactacctttcgttctgtagc	25	53.2	-27.8	35.4	36.0	86	85	192/75	56

Table S1. Analysis of universal SNPs for human identification and the lactose intolerance (the MCM6 gene) by ASQ method.

SNP	Allele	ID	Primer Info	Sequence (5'-3')	nt	Tm (°C)*	dG (kcal/mol)	Tm 3'end (°C)	GC (%)	LC (%)	PQ (%)	Fragment Size (bp)/Tm (°C)
rs560681	A/G	5665	rs560681:1F_1_479-500_ASP1	CCAGCTGAACGGTGCTCACggtcctgtgacctgagtaaaCA	22	55.7	-26.8	30.3	50.0	82	82	
		5666	rs560681:1F_1_476-500_ASP2	CCAGCTGAACGGTTGCGACcaaggtcctgtgacctgagtaaaTA	25	55.8	-29.1	25.0	44.0	86	78	
		5667	rs560681:R_593-615	atggagaagcagtgaaaatcacc	23	55.2	-27.4	30.0	43.5	85	80	137/80
rs7520386	A/C/G	5668	rs7520386:1F_1_481-500_ASP1	CCAGCTGAACGGTGCTCACtgtgtttggtgagctgtaCG	20	54.8	-25.1	37.3	50.0	79	69	
		5669	rs7520386:1F_1_478-500_ASP2	CCAGCTGAACGGTTGCGACtgatgtgtttggtgagctgtaTG	23	55.2	-27.5	32.7	43.5	73	63	
		5670	rs7520386:R_583-603	gcaatggaggatccagcactt	21	56.8	-26.7	35.2	52.4	87	87	123/81
rs1490413	A/C/G	5671	rs1490413:1F_1_483-500_ASP1	CCAGCTGAACGGTGCTCACctgaggccagccagttCT	18	55.5	-23.8	38.5	61.1	78	78	
		5672	rs1490413:1F_1_482-500_ASP2	CCAGCTGAACGGTTGCGACtctgaggccagccagttTT	19	55.2	-24.6	36.3	52.6	76	66	
		5673	rs1490413:R_623-650	ctgtcactattattgtcagaatttggct	28	55.7	-31.5	32.4	35.7	80	80	168/79
rs1454361	A/T	5674	rs1454361:1F_1_480-500_ASP1	CCAGCTGAACGGTGCTCACgaaatacaccctgagctgcAT	21	54.5	-25.6	39.5	47.6	85	80	
		5675	rs1454361:1F_1_479-500_ASP2	CCAGCTGAACGGTTGCGACggaaatacaccctgagctgcTT	22	56.6	-27.4	39.3	50.0	88	73	
		5676	rs1454361:R_625-643	cagccctgtcaacctgaaa	19	54.0	-23.9	31.8	52.6	81	81	164/79
rs727811	A/C	5677	rs727811:1F_1_480-500_ASP1	CCAGCTGAACGGTGCTCACccggaacttcaacgacttaCA	21	54.5	-25.7	32.2	47.6	79	79	
		5678	rs727811:1F_1_479-500_ASP2	CCAGCTGAACGGTTGCGACaccggaacttcaacgacttaAA	22	54.0	-26.3	29.0	40.9	80	80	
		5679	rs727811:R_581-604	tgtattagtggccttgaggatcac	24	56.0	-28.5	31.6	45.8	95	91	125/75
rs2111980	T/C	5680	rs2111980:1F_1_483-500_ASP1	CCAGCTGAACGGTGCTCACcttggcagcatccttcGA	18	54.0	-23.3	37.4	55.6	83	83	
		5681	rs2111980:1F_1_480-500_ASP2	CCAGCTGAACGGTTGCGACcatcttggcagcatccttcAA	21	55.1	-25.9	33.9	47.6	77	77	
		5682	rs2111980:R_626-648	ctccaggctgtgactaagtcatt	23	56.0	-27.8	26.4	47.8	98	80	166/83
rs1454361	A/T	5683	rs1454361:1F_1_480-500_ASP1	CCAGCTGAACGGTGCTCACgaaatacaccctgagctgcAT	21	54.5	-25.6	39.5	47.6	85	80	
		5684	rs1454361:1F_1_479-500_ASP2	CCAGCTGAACGGTTGCGACggaaatacaccctgagctgcTT	22	56.6	-27.4	39.3	50.0	88	73	
		5685	rs1454361:R_563-582	gggtgaggcctgagttatcc	20	56.1	-25.4	30.2	60.0	84	84	103/77
rs4988235 (MCM6 gene)	C/G/T	5686	rs4988235:1F_1_475-500_ASP1	CCAGCTGAACGGTGCTCACggcaatacagataagataatgtagCC	26	53.7	-28.6	27.6	38.5	77	77	
		5687	rs4988235:1F_1_473-500_ASP2	CCAGCTGAACGGTTGCGACctggcaatacagataagataatgtagTC	28	53.7	-30.0	22.9	35.7	80	80	
		5688	rs4988235:1F_1_475-500_ASP3	CCAGCTGAACGGTGTGCGAggcaatacagataagataatgtagGC	26	53.7	-28.6	27.6	38.5	73	68	
		5689	rs4988235:R_580-601	cactgacctatcctcgtggaat	22	55.4	-26.7	36.6	50.0	90	90	127/77

* T_m calculated for oligonucleotide concentration of 250 nM, 50 mM K^+, in the absence of Mg^{2+}.
